# A nomogram based on cuproptosis-related genes predicts 7-year relapse-free survival in patients with estrogen receptor-positive early breast cancer

**DOI:** 10.3389/fonc.2023.1111480

**Published:** 2023-05-12

**Authors:** Yu Fan, Chuanxu Luo, Yu Wang, Zhu Wang, Chengshi Wang, Xiaorong Zhong, Kejia Hu, Yanping Wang, Donghao Lu, Hong Zheng

**Affiliations:** ^1^ Breast Center and Multi-omics Laboratory of Breast Diseases, West China Hospital, Sichuan University, Chengdu, China; ^2^ West China Biomedical Big Data Center, West China Hospital, Sichuan University, Chengdu, China; ^3^ Institute of Environmental Medicine, Karolinska Institutet, Stockholm, Sweden

**Keywords:** breast cancer, cuproptosis-related genes, nomogram, ER positive, RFS

## Abstract

**Introduction:**

Excess copper induces cell death by binding to lipoylated components of the tricarboxylic acid cycle. Although a few studies have examined the relationship between cuproptosis-related genes (CRGs) and breast cancer prognosis, reports on estrogen receptor-positive (ER+) breast cancer are lacking. Herein, we aimed to analyze the relationship between CRGs and outcomes in patients with ER+ early breast cancer (EBC).

**Methods:**

We conducted a case-control study among patients with ER+ EBC presenting poor and favorable invasive disease-free survival (iDFS) at West China Hospital. Logistic regression analysis was performed to establish the association between CRG expression and iDFS. A cohort study was performed using pooled data from three publicly available microarray datasets in the Gene Expression Omnibus database. Subsequently, we constructed a CRG score model and a nomogram to predict relapse-free survival (RFS). Finally, the prediction performance of the two models was verified using training and validation sets.

**Results:**

In this case-control study, high expression of *LIAS*, *LIPT1*, and *ATP7B* and low *CDKN2A* expression were associated with favorable iDFS. In the cohort study, high expression of *FDX1*, *LIAS*, *LIPT1*, *DLD*, *PDHB*, and *ATP7B* and low *CDKN2A* expression were associated with favorable RFS. Using LASSO-Cox analysis, a CRG score was developed using the seven identified CRGs. Patients in the low CRG score group had a reduced risk of relapse in both training and validation sets. The nomogram included the CRG score, lymph node status, and age. The area under the receiver operating characteristic (ROC) curve (AUC) of the nomogram was significantly higher than the AUC of the CRG score at 7 years.

**Conclusions:**

The CRG score, combined with other clinical features, could afford a practical long-term outcome predictor in patients with ER+ EBC.

## 1 Introduction

In 2020, breast cancer (BC), for the first time, surpassed lung cancer as the most commonly diagnosed cancer. Overall, an estimated 2.3 million new cases of BC have been diagnosed (1). According to data on BC pathology among Asian women, 52–76% of patients with BC have estrogen receptor (ER)-positive (ER+) subtypes (2). Expression of ER has been associated with a favorable prognosis and can predict the efficacy of endocrine therapies, including aromatase inhibitors and tamoxifen (3). However, nearly one-quarter of patients with ER + early BC (EBC) experience relapse within 10 years (4). Approximately half of all relapses occur after five years of adjuvant endocrine therapy (5). Although the clinical stage, combined with other features like ki67 and differentiation grade, can afford a preliminary assessment of prognosis, additional molecular markers are needed to construct an exemplary long-term prognosis model. Furthermore, these molecular markers could help identify more effective therapeutic targets.

The copper-dependent regulation of cell death is distinct from known death mechanisms and depends on mitochondrial respiration in human cells (6). Copper directly binds to lipoylated components of the tricarboxylic acid (TCA) cycle, resulting in lipoylated protein aggregation (7) and subsequent iron-sulfur cluster protein loss, thereby inducing proteotoxic stress (8) and, ultimately, cell death (9). The regulators essential for cuproptosis include two components, the lipoic acid (LA) pathway (*FDX1*, *LIAS*, *LIPT1*, and *DLD*) and the pyruvate dehydrogenase (PDH) complex (*DLAT*, *PDHA1*, *PDHB*, *MTF1*, *GLS*, and *CDKN2A*) (9).

Research on cuproptosis and its relationship with cancer remains in its early stages of development. Correlations between cuproptosis-related genes (CRGs) and prognosis have been reported in patients with renal carcinoma (10), head and neck cancer (11), melanoma (12), glioma (13), and BC. However, data mining on BC and CRGs currently involves The Cancer Genome Atlas (TCGA) database with a relatively short follow-up period, and molecular subtype analysis is required. The relationship between CRGs and the long-term prognosis of ER+ EBC remains unclear.

Herein, we first suggested a correlation between the CRG profile and invasive disease-free survival (iDFS) or relapse-free survival (RFS) in patients with ER+ EBC by performing a case-control study at our hospital and a cohort study from publicly available datasets. In the case-control study performed at the West China Hospital (WCH), high expression of *LIAS*, *LIPT1*, and *ATP7B* and low expression of *CDKN2A* significantly reduced the odds ratio (OR) of iDFS in patients with ER+ EBC. For validation, we used pooled data from three publicly available microarray studies (GSE42568, GSE9195, and GSE20685). In this cohort, high expression of *LIAS*, *LIPT1*, and *ATP7B* and low expression of *CDKN2A* significantly reduced the hazard ratio (HR) of relapse of ER+ EBC. Moreover, high expression of *FDX1* and *DLD*, two molecules of the LA pathway, and *PDHB* of the PDH complex significantly reduced the HR for relapse. We then constructed a CRG score model in the training set, which confirmed that a high CRG score could significantly increase the risk of relapse in both training and validation datasets. The nomogram comprising CRG score, lymph node status, and age had an increased area under the receiver operating characteristics (ROC) curve (AUC) at 7 years when compared with that of the CRG score alone.

In brief, a novel nomogram comprising the CRG score and clinical features could predict the 3-, 5-, and 7-year relapse risks of ER+ EBC. Targeted enhancement of cuproptosis may be a potential therapeutic strategy for patients with ER+ BC.

## 2 Materials and methods

### 2.1 WCH patients

Since 1989, patients with BC have been enrolled in the Breast Cancer Management Information System of WCH, Sichuan University (14). Physicians collected medical records, pathological diagnoses, and treatment information. Between January 2008 and April 2018, 7,784 females diagnosed with non-metastatic invasive BC were prospectively followed up for clinical outcomes (15). Patients with freshly frozen tumor and germline samples, including blood or normal breast tissue, available during primary surgery were eligible for study inclusion (N = 1462)

### 2.2 Case-control study of WCH

A matched extreme case-control design was employed (16). Female patients diagnosed with EBC (stages I-III) who were assessed for any iDFS endpoint at 7 years after diagnosis were grouped into cases, and patients who survived without any iDFS endpoint for at least 7 years were grouped into controls. One control was randomly selected per case and individually matched to the case of molecular subtype classified according to the St. Gallen Consensus 2013, as previously described (15). Any local or regional relapse, distant metastasis, new primary tumors from any site, cancer-specific death, or death from other causes were defined as iDFS endpoints. Subsequently, samples from 222 patients were RNA sequenced. Only ER+ samples were selected for further analysis, and ER- samples were excluded. Sixty-three patients were included in the case group and 62 in the control group. This study was approved by the Clinical Test and Biomedical Ethics Committee of the WCH, Sichuan University (No. 2019-16).

### 2.3 Pooled data from three mRNA expression datasets, GSE42568, GSE9195, and GSE20685

To verify the correlation between cuproptosis and patient outcomes, we selected three GSE datasets (GSE42568, GSE9195, and GSE20685) from the NCBI for Biotechnology Information Gene Expression Omnibus (GEO). The data met the following criteria: 1. Affymetrix Human Genome U133 Plus 2.0 Array; 2. provided ER status, tumor size, T stage, lymph node status, N stage, and age of patients with BC; 3. comprised at least 70 samples; 4. employed RFS as the endpoint; 5. at least 80% of the relapse-free patients were followed up for more than 5 years. In total, 508 samples from the three datasets were downloaded from “https://www.ncbi.nlm.nih.gov/geo/”. We excluded 164 samples owing to metastasis at diagnosis or ER-. Overall, 344 patients were included in the subsequent analysis.

### 2.4 RNA sequencing data preparation

In the case-control study, RNA sequencing of frozen tumor tissues was performed using the Illumina NovaSeq S6000 platform. After quality control, reads were mapped to the reference genome using Hisat2 v2.0.5, as previously described (15). To calculate the fragments per kilobase of exons per million reads (FPKM) for gene i, the following formula was used:


$$\text{FPKMi}=\frac{\text{qi}}{\text{li}*\Sigma \text{jqj}}*10^{9}$$

where qi is the raw read count, li is the gene length, and Σjqj corresponded to the total number of mapped reads (17). All FPKM data were then log2(x+1) transformed.

### 2.5 Microarray data preparation

The expression matrixes and clinical data for GSE42568, GSE9195, and GSE20685 were downloaded from “https://www.ncbi.nlm.nih.gov/geo”. The R package limma (v3.48.1) was used to remove batch effects of these three GSE datasets, and principal component analysis (PCA) of each sample was performed before and after normalization. Each gene corresponded to a probe, except for *CDKN2A*, which corresponded to three probes. The probe with the highest normalized intensity averaged over all samples, was used to represent the expression level of *CDKN2A*. RFS endpoints were identical to iDFS endpoints, except for the occurrence of invasive contralateral BC, secondary primary invasive cancer, and contralateral ductal carcinoma *in situ* (18).

### 2.6 Association between CRGs and iDFS in the case-control study

We analyzed 13 CRGs from earlier studies: *FDX1*, *LIAS*, *LIPT1*, *DLD*, *DLAT*, *PDHA1*, *PDHB*, *MTF1*, *GLS*, *CDKN2A*, *SLC31A1*, *ATP7A*, and *ATP7B* (9, 19). Univariate and multivariate logistic regression analyses were performed to determine the association between individual CRG expression levels and iDFS endpoints. Pearson or Spearman correlation coefficient (r) was used for measuring the relationship between individual CRGs and clinicopathological features, including T stage, N stage, ki67, grade, and HER2 status in the WCH cohort.

### 2.7 Construction and validation of a prognostic CRG score in the cohort study

Univariate and multivariate Cox analyses of RFS were performed to screen for individual CRGs with prognostic values in the pooled GSE dataset. Kaplan-Meier survival analysis was used to estimate the RFS between the high- and low- CRGs expression groups. The “survminer” R package (V0.4.9) provided a cut-off for facilitating survival analysis. The 344 enrolled patients were randomly divided into two sets (7:3), with 241 and 103 patients in the training and validation sets, respectively. In the training set, CRGs with *P*<0.05 in the multivariate Cox regression were subjected to LASSO-penalized Cox regression analysis to construct a prognostic CRG score model using the “glmnet” R package (v4.1). The hyperparameter lambda (λ) was chosen based on tenfold cross-validation with the slightest mean squared error. Patient CRG scores were calculated according to the normalized expression levels of each gene and corresponding regression coefficients. The LASSO-penalized Cox regression formula is as follows:


$$\text{CRGs.score}=\sum_{\text{i}=1}^{\text{n}}\left({\text{β}}_{i}\times \exp\text{ression}({\text{gene}}_{\text{i}})\right)$$

(20)β_i_ represented the corresponding coefficient of a specific gene, and the expression(gene_i_) indicates the expression level of the corresponding gene. The CRGs score for each patient could be calculated according to the formula. ROC curve analysis was used to assess the performance of the CRG score using the R package “timeROC” (V0.4). Univariate and multivariate Cox analyses and Kaplan-Meier survival analysis were performed to verify the association between CRGs score and RFS in the training and validation sets.

### 2.8 Construction and validation of a prognostic nomogram based on CRGs score

A nomogram model predicting RFS was developed based on CRG scores and other clinical features, including lymph node status and age, in the training set using the R package “rms” (V6.3). Univariate Cox and Kaplan-Meier survival analyses were performed to verify the relationship between the nomogram points and RFS. The ROC curve assessed the performance of the nomogram model using the “timeROC” package in both the training and validation sets. A comparison of ROCs was performed between the CRG score and nomogram points using the “compare” function in the “timeROC” package.

Statistical analyses were performed using R software (V4.1.0). Statistical significance was set at *P*< 0.05.

## 3 Results

### 3.1 Study design

**Figure 1:**
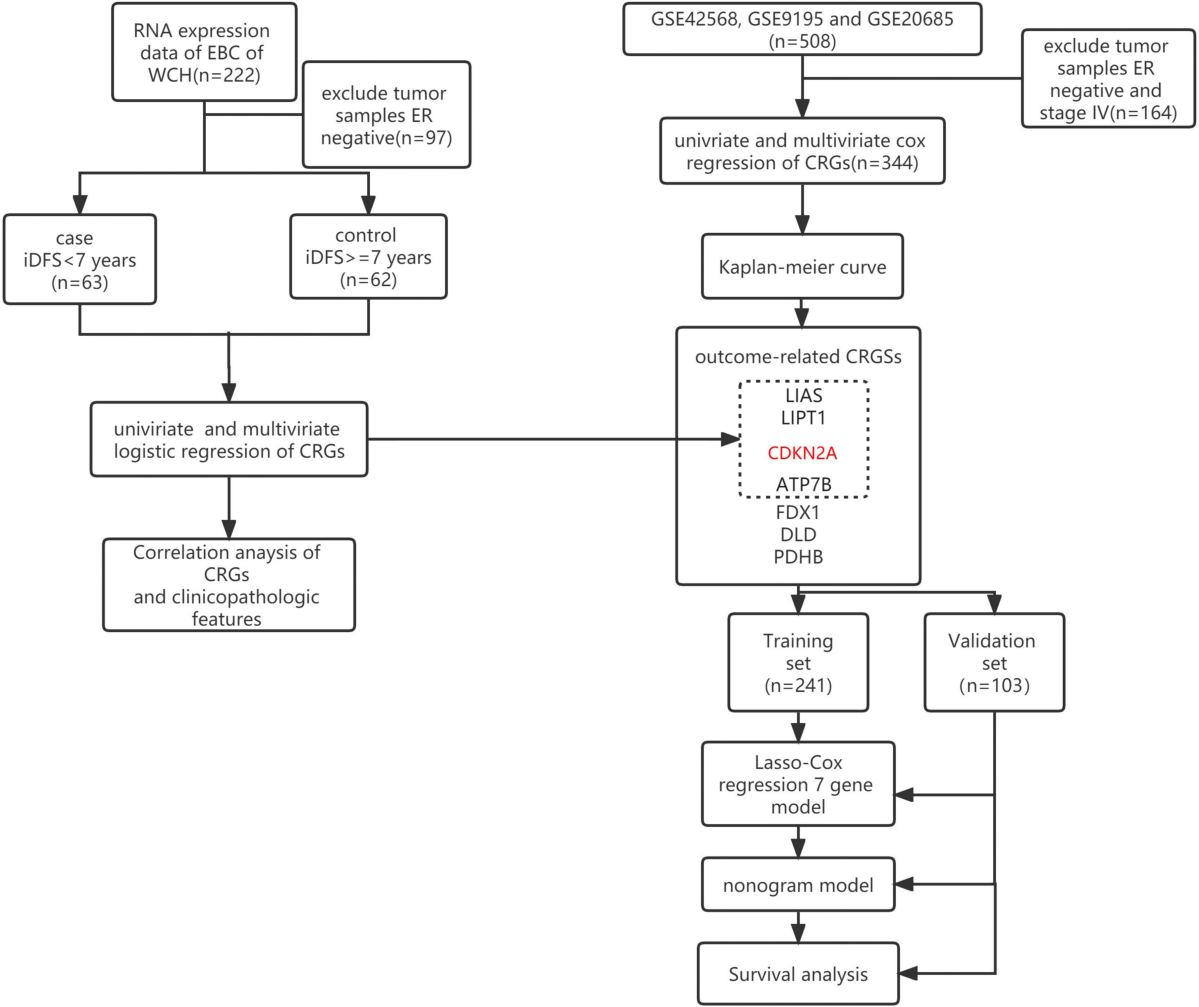
Flow diagram of the patient selection and study design. The red-marked gene represents the gene that negatively hit cuproptosis. Black-marked genes represent the genes that positively hit cuproptosis. WCH, West China Hospital; iDFS, invasive disease-free survival; ER, estrogen receptor; CRGs, cuproptosis-related genes.

**Figure 1** presents the flow chart of the study. Our study used two datasets and two study designs to demonstrate the association between CRGs and iDFS or RFS in patients with ER+ EBC. The genes in dotted boxes represent overlapping genes associated with iDFS or RFS in both datasets.

### 3.2 Characteristics of patients in the case-control study

In the case-control study, 63 patients who experienced endpoint events within 7 years were grouped into cases, and the other 62 patients were grouped into controls. The two groups had no significant differences in menopause, age, T stage, N stage, grade, ki67, progesterone receptor status, or treatment, according to the chi-square test. The control group had a higher proportion of HER2-positive patients, given that the HER2 status of 8 patients was uncertain in the case group. The average iDFS and overall survival (OS) were 127.1 ± 15.2 and 127.1 ± 15.2 months in the control group and 31.4 ± 22.5 and 62.5 ± 35.2 months in the case group, respectively (**Table 1**).

**Table 1:** Clinical and pathological features of 125 estrogen receptor positive invasive breast cancer patients in West China Hospital.

Features	levels	Control (N = 62)	Case(N=63)	p
menopause	No	35 (56.5%)	32 (50.8%)	0.649
	Yes	27 (43.5%)	31 (49.2%)	
age	<40y	13 (21%)	8 (12.7%)	0.319
	≥40y	49 (79%)	55 (87.3%)	
T stage	1	23 (37.1%)	12 (19%)	0.252
	2	34 (54.8%)	43 (68.3%)	
	3	2 (3.2%)	3 (4.8%)	
	4	2 (3.2%)	4 (6.3%)	
	unknown	1 (1.6%)	1 (1.6%)	
N status	0	31 (50%)	20 (31.7%)	0.055
	1	19 (30.6%)	18 (28.6%)	
	2	8 (12.9%)	13 (20.6%)	
	3	4 (6.5%)	12 (19%)	
grade	1	0 (0%)	1 (1.6%)	0.114
	2	23 (37.1%)	15 (23.8%)	
	3	31 (50%)	43 (68.3%)	
	unknown	8 (12.9%)	4 (6.3%)	
ki67	≤10%	7 (11.3%)	4 (6.3%)	0.51
	>10%	55 (88.7%)	59 (93.7%)	
PR	negative	54 (87.1%)	49 (77.8%)	0.257
	positive	8 (12.9%)	14 (22.2%)	
HER2 status	negative	42 (67.7%)	36 (57.1%)	0.014*
	positive	20 (32.3%)	19 (30.2%)	
	uncertain	0 (0%)	8 (12.7%)	
chemotherapy	No	1 (1.6%)	4 (6.3%)	0.371
	Yes	61 (98.4%)	59 (93.7%)	
radiotherapy	No	36 (58.1%)	36 (57.1%)	1
	Yes	26 (41.9%)	27 (42.9%)	
Herceptin	No	57 (91.9%)	58 (92.1%)	1
	Yes	5 (8.1%)	5 (7.9%)	
OS_bin	0	62 (100%)	33 (52.4%)	<.001
	1	0 (0%)	30 (47.6%)	
iDFS_month	Mean ± SD	127.1 ± 15.2	31.4 ± 22.5	<.001
OS_month	Mean ± SD	127.1 ± 15.2	62.5 ± 35.2	<.001

### 3.3 CRG expression associated with iDFS in the case-control study

**Figure 2:**
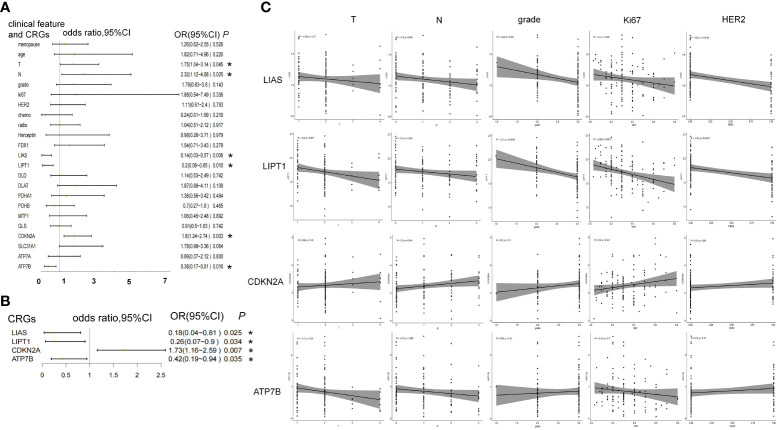
The association between CRGs and iDFS endpoints in WCH case-control study. **(A)** Univariate and **(B)** multivariate logistic regression analyses of clinical features and CRGs for association with iDFS in the WCH cohort. **(C)** The relation of CRGs and the T stage, N stage, grade, ki67 and HER2 status. *, a p -value less than 0.05.

In the case-control study, the higher expression level of *LIAS* (OR = 0.14, 95% confidence interval [CI] 0.03–0.57, *P* = 0.008), *LITP1* (OR = 0.2, 95%CI 0.06–0.65, *P* = 0.01), and *ATP7B* (OR = 0.38, 95%CI 0.17–0.81, *P* = 0.016) was associated with a lower risk of iDFS endpoints. However, higher T stage (OR = 1.75, 95%CI 1.04–3.14, *P* = 0.045), N stage (OR = 2.32, 95%CI 1.12–4.88, *P* = 0.025), and *CDKN2A* expression (OR = 1.8, 95%CI 1.24–2.74, *P* = 0.003) were associated with a higher risk of iDFS endpoints (**Figure 2A**; **Supplementary Table 1**). Menopause, age, grade, ki67, HER2 status, chemotherapy, radiotherapy, trastuzumab, and other CRGs showed no association with iDFS endpoints (**Figure 2A**). Adjusted for T stage and N stage, *LIAS* (OR = 0.18, 95%CI 0.04–0.81, *P* = 0.025), *LIPT1* (OR = 0.26, 95%CI 0.07–0.9, *P* = 0.034), *CDKN2A* (OR = 1.73, 95%CI 1.16–2.59, *P* = 0.007), and *ATP7B* (OR = 0.42, 95%CI 0.19–0.94, *P* = 0.035) were still associated with iDFS endpoints (**Figure 2B**; **Supplementary Table 2**).

Furthermore, we noted the relationship between these four genes and other clinicopathological features. *LIAS* was negatively associated with N stage (R = -0.18, *P* = 0.041), tumor grade (R = -0.24, *P* = 0.012), ki67 (R = -0.2, *P* = 0.029), and HER2 expression (R = -0.35, *P* = 9E-5). *LITP1* was negatively associated with T stage (R = -0.2, *P* = 0.027), N stage (R = -0.16, *P* = 0.066, borderline significance), grade (R = -0.31, *P* = 0.001), ki67 (R = -0.28, *P* = 0.002), and HER2 levels (R = -0.32, *P* = 0.0004). *CDKN2A* expression was positively associated with N stage (R = 0.19, *P* = 0.034) and ki67 (R = 0.18, *P* = 0.047). *ATP7B* expression was negatively associated with T stage (R = -0.17, *P* = 0.06, borderline significance) (**Figure 2C**).

### 3.4 Expression of CRGs associated with RFS in the cohort study

**Figure 3:**
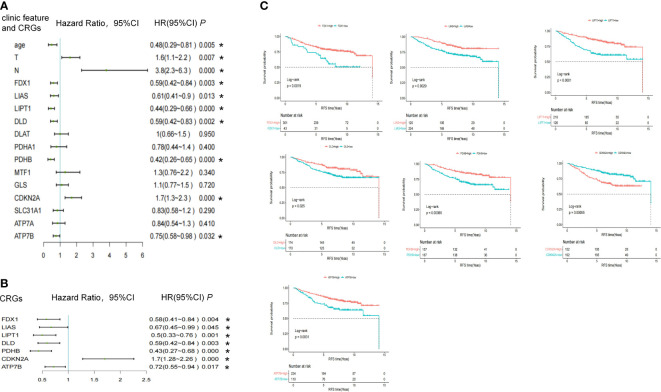
The association between CRGs and RFS endpoints in the pooled cohort study. **(A)** Univariate and **(B)** multivariate cox regression of clinical features and CRGs in pooled GSE data. **(C)** Kaplan-Meier survival analysis of CRGs. *, a p -value less than 0.05.

In total, 344 patients were diagnosed with stage I-III ER-positive BC using the GSE42568, GSE9195, and GSE20685 databases. **Supplemental Figure 1** shows the PCA of each sample before and after normalization using the R package “limma.” Tumor size (HR = 1.6, 95%CI 1.1–2.2, *P* = 0.007) and lymph node status (HR = 3.8, 95%CI 2.3–6.3, *P* = 2.6E-07) were risk factors for relapse, and older age (HR = 0.48, 95%CI 0.29-0.81, *P* = 0.006) was a protective factor against relapse. Of identified CRGs, expression levels of *LIAS* (HR = 0.61,95%CI 0.41–0.9, *P* = 0.013), *LITP1* (HR = 0.44, 95%CI 0.29–0.66, *P* = 7.40E-05), *CDKN2A* (HR = 1.7, 95%CI 1.3–2.3, *P* = 0.0001), *ATP7B* (HR = 0.75, 95%CI 0.58–0.98, *P* = 0.032), *FDX1* (HR = 0.59, 95%CI 0.42–0.84, *P* = 0.003), *DLD* (HR = 0.59, 95%CI 0.42–0.83, *P* = 0.002), and *PDHB* (HR = 0.42, 95%CI 0.26-0.65, *P* = 0.0002) were associated with RFS (**Figure 3A**; **Supplementary Table 3**). After adjusting for tumor size, lymph node status, and age, these seven genes were still associated with the risk of relapse (**Figure 3B**; **Supplementary Table 4**). The high expression of *LIAS*, *LITP1*, *ATP7B*, *FDX1*, *DLD*, and *PDHB* and the low expression of *CDKN2A* were associated with longer RFS, as determined by the Kaplan-Meier curve (**Figure 3C**). Collectively, five genes positively affected cuproptosis, and one copper transporter gene decreased the risk of relapse. However, one gene negatively impacting cuproptosis may also increase the risk of relapse.

### 3.5 Construction CRGs score model in GSE dataset and validation

**Figure 4:**
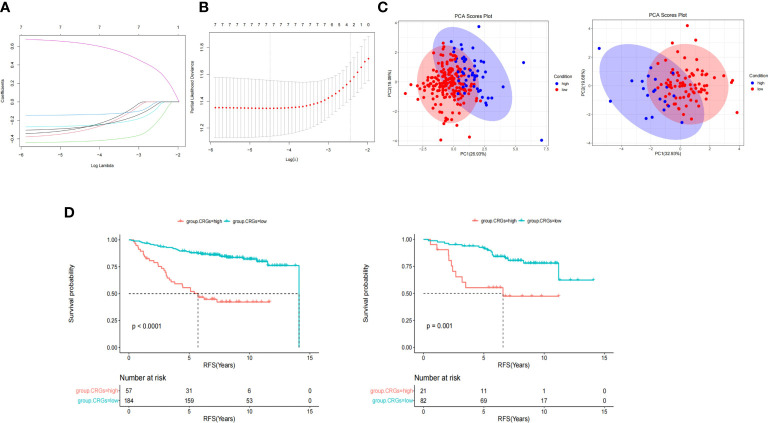
CRGs score model construction and survival analyses. **(A)** LASSO cox regression analysis was used to establish a prognostic model in the training group based on the expression profiles of 7 CRGs. **(B)** 7 gene signatures were determined based on the optimal value of λ. **(C)** Principal component analysis (PCA) showed patients in different CRGs score groups distribution. **(D)** Kaplan-Meier survival analysis of CRGs score in training (left) and validation set (right).

Enrolled patients (n = 344) were randomly divided into two sets (7:3): the training set (n = 241) and the validation set (n = 103). The training and validation sets showed no significant differences in clinical features or CRG expression (**Supplementary Table 5**). LASSO-Cox regression analysis was used to establish a prognostic model for the training set based on expression profiles of the seven genes (**Figure 4A**). Seven gene signatures were determined based on the optimal value (**Figure 4B**). The risk score was then calculated based on the coefficient of each gene as follows:

CRGs.score = -FDX1*0.283 -LIAS*0.314 -LIPT1*0.428 -DLD*0.139 -PDHB*0.257 +CDKN2A*0.635 -ATP7B*0.275

**Figure 5:**
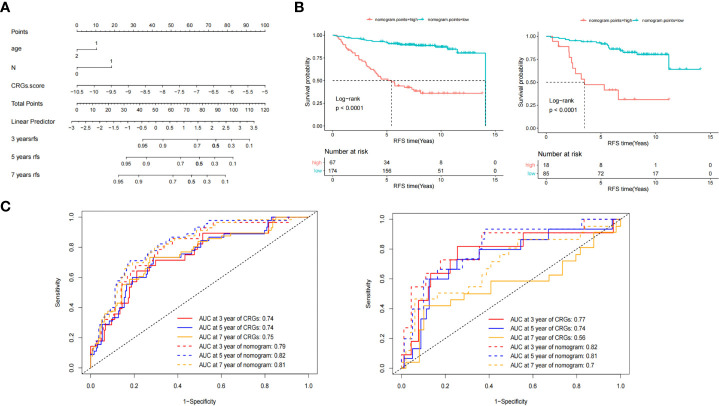
Nomogram model construction and survival analyses. **(A)** The Nomogram comprised the CRGs core, age, and lymph node status. **(B)** Kaplan-Meier survival analysis of nomogram points in training (left) and validation set (right). **(C)** The area under the curve (AUC) of receiver operating characteristics (ROC) of CRGs core and nomogram points model at 3-, 5- and 7- years in training (left) and validation (right) set.

According to the cut-off value of the CRG score calculated using the R package “survminer,” patients were divided into the high CRG score group and the low CRG score group in the training and validation set. PCA revealed that patients in the different CRG score groups were distributed in two directions (**Figure 4C**). As shown in **Figure 4D**, the median survival time in the low and high CRG score groups was 14.1 and 5.73 years, respectively, in the training set. The HR of the low CRG score group was 0.21 (95%CI 0.13–0.35, *P* = 1.1E-9) when compared with that of the high CRG score group. After adjustment for tumor size, lymph node status and age, the HR of low CRG score was 0.24 (95%CI 0.14–0.41, *P* = 1.07E-07). The AUC value was used to evaluate the predictive performance of the CRG score over time. For the training set, the AUC was 0.74 at 3 years, 0.74 at 5 years, and 0.75 at 7 years (**Figure 5C**, left, solid line).

In the validation set, the median survival time was not reached (NR) and 6.59 years for the low and high CRG score groups, respectively. The HR of the low CRG score group was 0.29 (95%CI 0.13–0.64, *P* = 0.002), determined by univariate Cox regression analysis, and 0.23 (95%CI 0.1–0.52, *P* = 0.0005) by multivariate Cox regression analysis. For the validation set, the AUC was 0.77 at 3 years, 0.74 at 5 years, and 0.56 at 7 years (**Figure 5C**, right, solid line).

### 3.6 Development and validation of a predictive nomogram based on CRG score

Multivariate Cox analysis of the CRG score, age, tumor size, and lymph node status was performed. Tumor size (*P* = 0.52) was not an independent prognostic factor. The CRG score (*P* = 1.80E-09) and lymph node status (*P* = 3.03E-06) were independent prognostic factors, while age reached borderline significance as an independent prognostic factor (*P* = 0.055).

Based on the multivariate analysis results, we developed a nomogram model as an easy-to-use tool (**Figure 5A**). As shown in (**Figures 5B, C**), the median RFS time of patients with low and high nomogram points in the training set were 14.1 and 5.42 years, respectively. The HR of patients with low nomogram points was 0.14 (95%CI 0.085–0.24, *P* = 2.4E-13). The AUC was 0.79 at 3 years, 0.82 at 5 years, and 0.81 at 7 years (**Figure 5C** left, dotted line). Compared with the CRG score, the AUC values of the nomogram at 5 (*P* = 0.002) and 7 years (*P* = 0.02) were significantly improved.

In the validation set, the median RFS time of patients with low and high nomogram points were NR and 3.5 years, respectively, and the HR of patients with low nomogram points was 0.17 (95%CI 0.079–0.38, *P* = 1.6E-05). The AUC was 0.82 at 3 years, 0.81 at 5 years, and 0.7 at 7 years, as shown in **Figure 5C** (right, dotted line). Compared with the CRG score, the AUC value of the nomogram points at 7 years (*P* = 0.03) significantly improved.

## 4 Discussion

In the present study, we employed two datasets and two study designs to demonstrate the association between CRGs and iDFS or RFS in patients with ER+ EBC. Considering patients from WCH and pooled GSE data, expression of *LIAS*, *LIPT1*, *CDKN2A*, and *ATP7B* correlated with patient endpoints, and the risk direction was consistent. In addition, three other CRGs, i.e., *FDX1*, *DLD*, and *PDHB*, were negatively associated with the risk of relapse in the GSE cohort. We then used pooled patients with GSEs to construct the CRG score model and a nomogram for RFS prediction. In the training and validation sets, the relapse risk of the high CRG score group, comprising the seven genes, was significantly higher than that of the low CRG score group. We used the CRG score combined with lymph node status and age to construct a nomogram and found that the RFS in the high-point group was significantly shorter than in the low-point group. The 7-year predicted AUC of the nomogram points was higher than that of the CRG score alone. The findings of the present study revealed the potential impact of CRGs on the clinicopathological features and prognosis of patients with ER+ EBC. Interestingly, five genes promoting cuproptosis were positively correlated with prognosis, and one gene inhibiting cuproptosis negatively correlated with prognosis, suggesting that cuproptosis may be a protective mechanism that reduces relapse in patients with ER+ EBC. Furthermore, high levels of LA pathway genes, including *FDX1*, *LIAS*, *LIPT1*, and *DLD*, correlated with prolonged RFS, suggesting that targeting the LA pathway in cuproptosis may be a potential therapeutic strategy in patients with ER+ BC.

Cu is a mineral nutrient, and a growing number of studies have confirmed the involvement of Cu in cell proliferation and death pathways (21). Given the intrinsic oxidized-reduced properties, Cu can be both beneficial and potentially toxic to cells. Cu is an essential cofactor for enzymes that mediate basic cellular functions, including mitochondrial respiration, antioxidant defense, and hormone and neurotransmitter biosynthesis. However, dysregulation of Cu storage can lead to oxidative stress and cytotoxicity (22, 23). First defined by Golub et al., cuproptosis is a new cell death pattern that reveals the critical mechanism through which CRGs regulate copper death (9). Copper ionophores, such as disulfide (24) and elesclomol (25) can induce oxidative stress by suppressing natural antioxidant systems, such as the mitochondria, thereby inducing copper death. However, research on cuproptosis remains in the early stages of development, and specific regulatory mechanisms in various cancers remain unexplored.

CRGs have been previously correlated with the prognosis of patients with renal carcinoma (10), head and neck cancer (11), melanoma (12), and glioma (13). Considering BC, Zhi et al. (26) analyzed the TCGA database and found that patients with high expression levels of *ATP7A*, *DBT*, *DLAT*, *DLD*, *GLS*, *PDHA1*, and *SLC31A1* had a poor prognosis. High expression levels of *ATP7B*, *LIPT1*, and *NLRP3* were associated with improved OS. Li et al. (27) found that expression of *SLC31A1*, *ATP7A*, *DLD*, *DLAT*, and *DBT* significantly increased the risk of death, as determined by analyzing the TCGA database. Li et al. (19) analyzed the TCGA database and found that DLAT, SLC31A1, ATP7A, and ATP7B expression levels were significantly related to the OS of patients with BC. Furthermore, Li et al. (28) found that SLC31A1 expression and its related pathway genes could potentially predict diagnosis, prognosis, and therapeutic response, as determined by analyzing the TCGA database. Sha et al. (29) analyzed a triple-negative subgroup of TCGA and found that high expression of *ATP7A*, *DLST*, and *LIAS* was associated with poor OS. Conversely, high expression levels of *LIPT1* and *PDHA1* indicated a good prognosis.

However, these studies have some limitations. First, the median follow-up time of patients without relapse in the TCGA database was 2.68 years for the ER+ subgroup. However, the relapse probability of ER+ patients within 5 years was the same as that after 5 years. Long-term follow-up of patients with ER+ BC is necessary. Second, analyses of BC subgroups, such as ER+ or HER2, are lacking in reported studies.

Herein, we used two independent long-term follow-up databases and two study designs to analyze the correlation between CRGs and outcomes in the ER+ subgroups and constructed a relapse prediction nomogram. Among patients enrolled at WCH, the control group had a median iDFS of 10.58 years. The median RFS of relapse-free patients was 7.3 years in the pooled GSE data. Considering the relationship between *DLD*, *LIAS*, and prognosis in TCGA, our findings contradict those reported in earlier studies, which may be attributed to the follow-up time and different molecular subgroups.

In our case-control study, the number of patients was limited. Therefore, we identified fewer prognostic genes than those in the pooled GSE data. In the cohort study, there were limited clinical features in public datasets that could be included in the model construction. Considering another limitation of our study, OS was not used as an endpoint.

In conclusion, our study indicates that high expression of positive hit genes (*FDX1, LIAS, LIPT1, DLD, PDH1*) and a copper-transporting gene (*ATP7B*) and low expression of negative hit genes (*CDKN2A*) related to cuproptosis can reduce the risk of iDFS or RFS in patients with ER+EBC. In addition, the constructed prognostic nomogram model had good predictive value for 7-year RFS of patients with ER+EBC.

## Data availability statement

The raw data supporting the conclusions of this article will be made available by the authors, without undue reservation.

## Ethics statement

The study involving human participants was conducted in accordance with the Declaration of Helsinki and was reviewed and approved by the Clinical Test and Biomedical Ethics Committee of West China Hospital, Sichuan University (No. 2019-16). The patients/participants provided their written informed consent to participate in this study

## Author contributions

YF: Literature review, statistical analysis and interpretation of data, drafting of the manuscript, review of the manuscript for important intellectual content and final approval of the version to be submitted. YW, ZW, CW, CL and KH: Collection, preservation, sorting and delivery of frozen specimens for sequencing and approval of the version to be submitted. XZ: Clinical data collection and collation, revision and approval of the version to be submitted. HZ, DL and YPW: Study concept, review of the manuscript for important intellectual content and final approval of the version to be submitted. All authors contributed to the article and approved the submitted version.
