# Supplementary material for: A nomogram based on cuproptosis-related genes predicts 7-year relapse-free survival in patients with estrogen receptor-positive early breast cancer
Source: Front Oncol. 2023 May 12;13:1111480. doi: 10.3389/fonc.2023.1111480 (PMC10213626; doi:10.3389/fonc.2023.1111480)
Supplement: Supplementary file 1 [file Image_1.pdf]

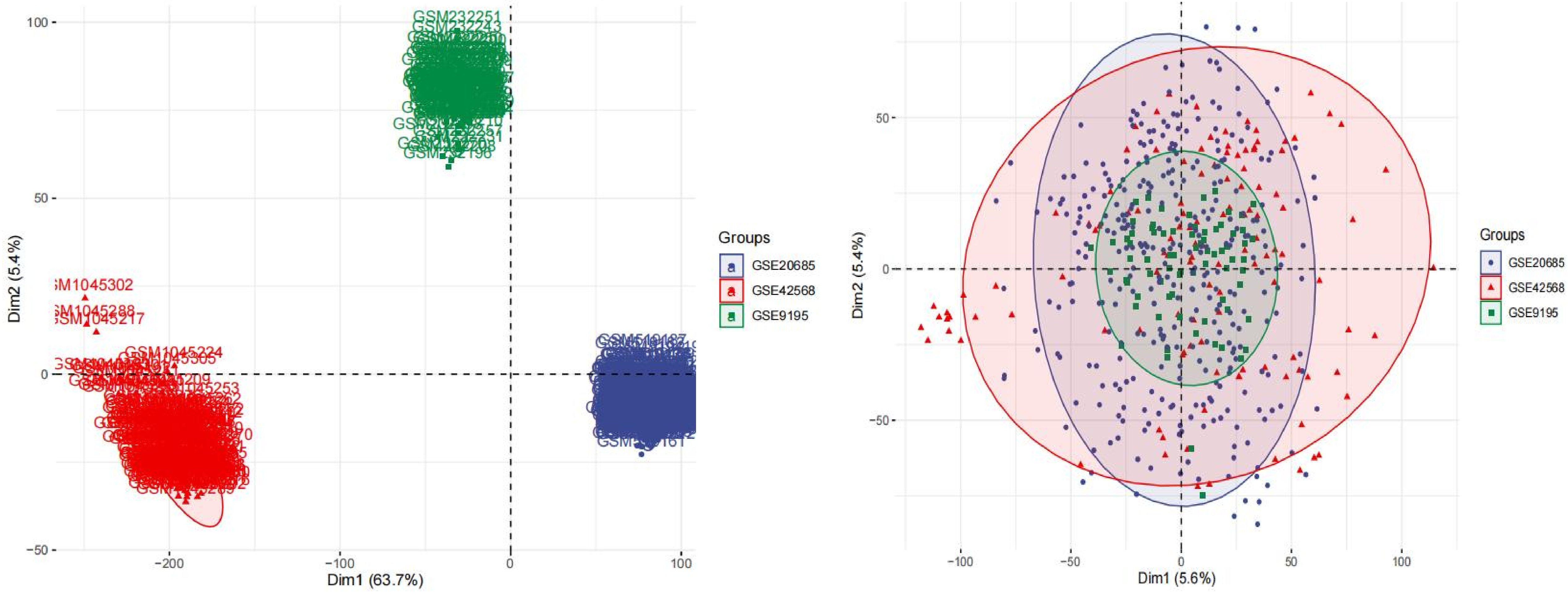

Before

After

Supplemental figure 1. The principal component analysis (PCA) of each samples before and after normalization using the R package “limma”.
